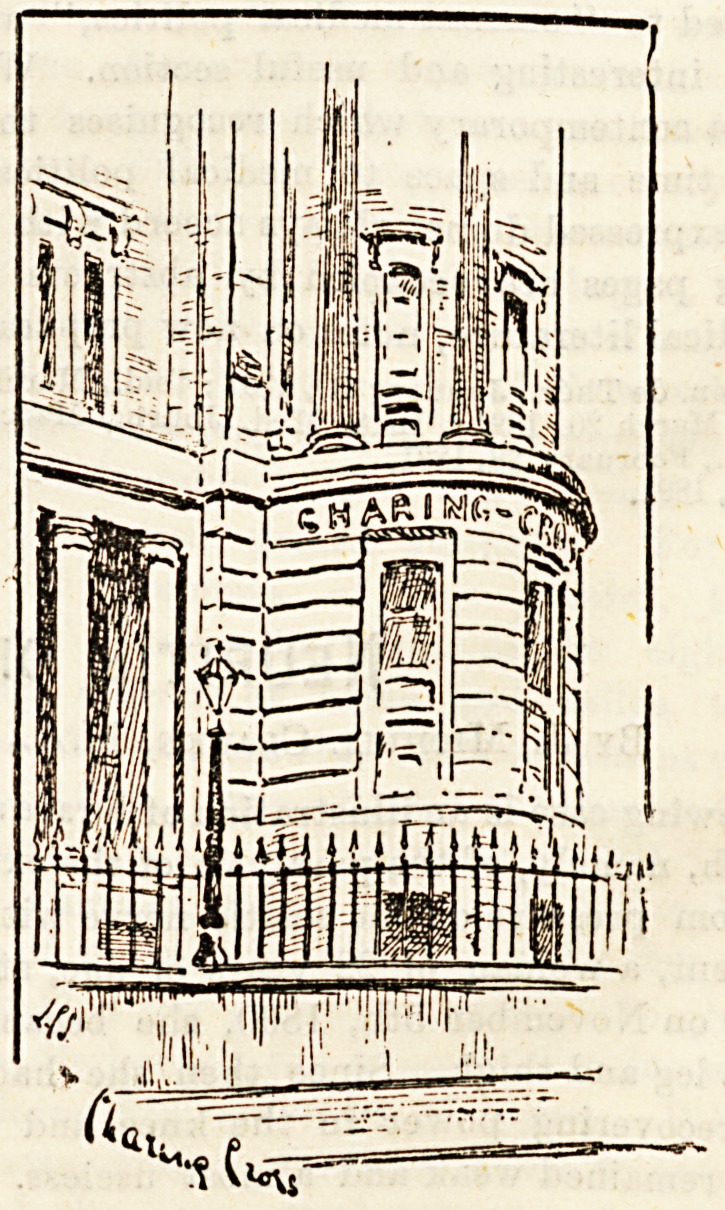# Portals of Health

**Published:** 1891-06-06

**Authors:** 


### Portals of Health.

When Sunday after Sunday we murmur, in that prayer for all sorts and conditions of men, the old familiar clause: "Especially those, &c.," our thoughts as a rule stray away to the sick and the suffering. Our imagination enters, without knocking, those portals of health dotted about the mighty metropolis in such numbers that the hospital-world now forms a section of the great city much larger than outsiders of the subject dream of. We can but dimly comprehend, though comforting ourselves that it exists, the magnitude of the blessed work that is going on, day by day, never relaxing for a single hour its tension, in these refuges for suffering mankind. It is beyond our mental grasp to conceive the vastness of this work of "relieving the several necessities" of those who are ailing; we can but try to master its comprehension piece by piece.

In wonder, we regard the machinery of, say, one hospital alone; the order; the discipline; the rigid wholesomeness, everything being dragged out into the honest light of day; the systematic routine throughout, which in itself is a medicine for the mind, at least; and then, we sit down and tell ourselves that all this organisation repeats itself nigh upon thirty times over, in the general hospitals alone of London, not to speak of the large number of special institutions.

Truly, then, we begin to realise that the hospital-world is one of the foundations of the life of London; and, presently, there comes stealing home to us the conviction of the importance of that foundation being a firm one. Common sense—setting apart all sentiment in the matter—points to that fact, and keeps its finger on it. Unless we put health of the body upon the very highest pedestal of importance, that foundation-stone will not be "well and truly laid." The very highest, we say, not forgetting that the sound mind must naturally be a sequence of the sound body; and, given a sound mind, it is there that the love of God will take up its abode, and the keynote of universal perfection be struck—perhaps faintly, being human as we are, but still with the true ring.

Such convictions are borne in upon most of us on that Sunday of the year's Sundays when the hospitals of our land plead, with outstretched hands, for that help which is their just right and due; that help which, if only for selfish reasons, we should think it a glad privilege to accord. When science, which God, in His goodness, is dealing out with a larger generosity each year, heals a brother-man of some cruel disease, and, setting him on his feet, sends him from those portals of health cured, the world is so much the more healthful, the cleaner, the more wholesome for you and me. And, in these days when mankind persists in crowding the towns of his own making, leaving empty and desolate the pure, God-made country, it is a matter that concerns and affects each and all to help on the great necessity of healing.

To the selfish, then, to those whose sole life-object is "getting and spending," we point out the wisdom of giving ungrudgingly of their abundance, and even of what can be ill-spared, for the sake of themselves, if for no others. But there are other hearts far different: men and women who, with glad alacrity, will pour out all they can, and even that little over which will be the truest test of their sincerity, for a higher motive, that is for the sake of their Master who went about "doing good," which must, by force of that example, be man's highest duty. By one and all let the pleading be taken to heart, and the good work of hastening on a "happy issue out of all their afflictions" to those for whom we are accustomed to pray—those who crowd the wards of our hospitals, to be watched and tended with skill and tender care, all through the livelong days of sickness and pain.

## Figures and Tables

**Figure f1:**
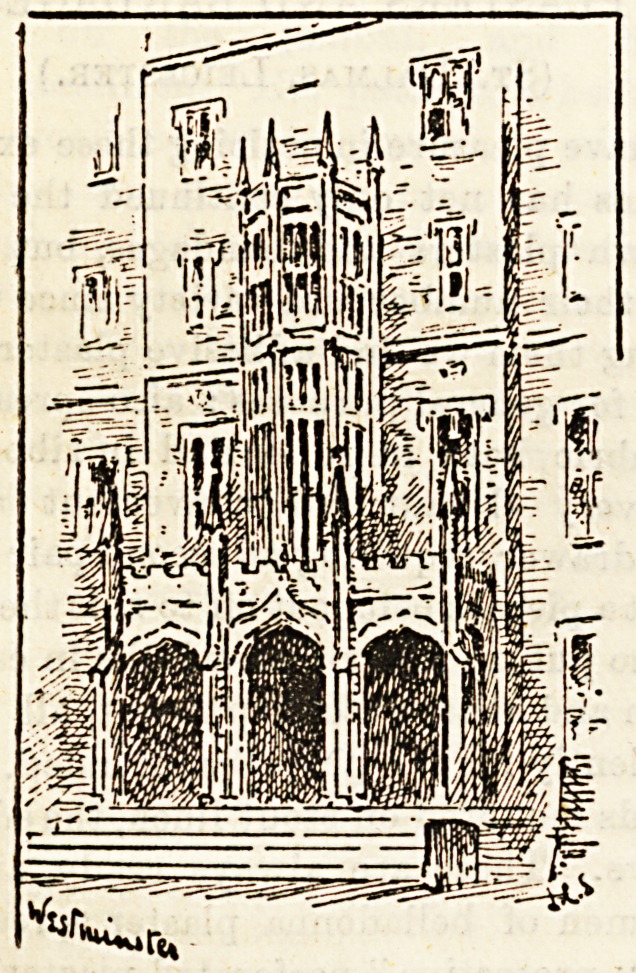


**Figure f2:**